# CD8 Lymphocyte Depletion Enhances the Latency Reversal Activity of the SMAC Mimetic AZD5582 in ART-Suppressed Simian Immunodeficiency Virus-Infected Rhesus Macaques

**DOI:** 10.1128/JVI.01429-20

**Published:** 2021-03-25

**Authors:** Maud Mavigner, Laura E. Liao, Alyssa D. Brooks, Ruian Ke, Cameron Mattingly, Nils Schoof, Julia McBrien, Diane Carnathan, Shan Liang, Thomas H. Vanderford, Mirko Paiardini, Deanna Kulpa, Jeffrey D. Lifson, Richard M. Dunham, Kirk A. Easley, David M. Margolis, Alan S. Perelson, Guido Silvestri, Ann Chahroudi

**Affiliations:** a Department of Pediatrics, Emory University School of Medicine, Atlanta, Georgia, USA; b Center for Childhood Infections and Vaccines, Children’s Healthcare of Atlanta and Emory University, Atlanta, Georgia, USA; c Theoretical Biology and Biophysics Group, MS-K710, Los Alamos National Laboratory, Los Alamos, New Mexico, USA; d Yerkes National Primate Research Center, Emory University, Atlanta, Georgia, USA; e AIDS and Cancer Virus Program, Frederick National Laboratory for Cancer Research, Frederick, Maryland, USA; f ViiV Healthcare, Research Triangle Park, North Carolina, USA; g University of North Carolina HIV Cure Center, Department of Medicine, UNC Chapel Hill, Chapel Hill, North Carolina, USA; h Department of Biostatistics and Bioinformatics, Emory University Rollins School of Public Health, Atlanta, Georgia, USA; i Emory Vaccine Center, Emory University, Atlanta, Georgia, USA; j Department of Pathology and Laboratory Medicine, Emory University School of Medicine, Atlanta, Georgia, USA; Icahn School of Medicine at Mount Sinai

**Keywords:** ART, CD8 lymphocyte depletion, SIV/HIV, SMAC mimetic, latency-reversing agents, rhesus macaques

## Abstract

Inducing latency reversal to reveal infected cells to the host immune system represents a potential strategy to cure human immunodeficiency virus (HIV) infection. In separate studies, we have shown previously that CD8^+^ T cells may contribute to the maintenance of viral latency and have identified a novel SMAC (second mitochondrial activator of caspases) mimetic/IAP (inhibitor of apoptosis protein) inhibitor (AZD5582) capable of reversing HIV/simian immunodeficiency virus (SIV) latency *in vivo* by activating the noncanonical (nc) NF-κB pathway. Here, we use AZD5582 in combination with antibody (Ab)-mediated depletion of CD8α^+^ cells to further evaluate the role of CD8^+^ T cells in the maintenance of viral latency. Six rhesus macaques (RMs) were infected with SIV_mac239_ and treated with antiretroviral therapy (ART) starting at week 8 postinfection. After 84 to 85 weeks of ART, all animals received a single dose of the anti-CD8α depleting Ab MT807R1 (50 mg/kg of body weight, administered subcutaneously [s.c.]), followed by five weekly doses of AZD5582 (0.1 mg/kg, administered intravenously [i.v.]). Following combined treatment with CD8α depletion and AZD5582, 100% of RMs experienced on-ART viremia levels above 60 copies per ml of plasma. In comparator groups of SIV-infected, ART-suppressed RMs treated with AZD5582 only or CD8α depletion only, on-ART viremia was experienced by 56% and 57% of the animals, respectively. Furthermore, the increased frequency of viremic episodes during the treatment period was greater in the group treated with CD8α depletion plus AZD5582 than in the other groups. Mathematical modeling of virus reactivation suggested that in addition to viral dynamics during acute infection, CD8α depletion influenced the response to AZD5582. This work suggests that the latency reversal induced by activation of the ncNF-κB signaling pathway with AZD5582 can be enhanced by CD8α^+^ cell depletion.

**IMPORTANCE** A favored approach to curing HIV infection aims at inducing viral expression using latency-reversing agents (LRAs) to allow the elimination of infected cells. Here, we tested a combination of two recently identified LRAs, the SMAC mimetic/IAP inhibitor AZD5582, which activates the noncanonical NF-κB pathway, and the antibody (Ab) MT807R1, which depletes CD8α^+^ cells, in SIV-infected rhesus macaques (RMs) on ART. Latency reversal, as defined by increased on-ART viremia, was observed in all six SIV-infected, ART-treated RMs that received this combined treatment. Furthermore, comparison of viral reactivation between these animals and groups of SIV-infected, ART-treated RMs treated with AZD5582 only or CD8α^+^ cell depletion only showed more frequent increases in viremic episodes when the two treatments were combined. This study provides additional evidence that CD8^+^ T cells may contribute to the maintenance of HIV/SIV latency on ART and potentially inhibit latency reversal during HIV cure approaches.

## Abstract (precis)

A favored approach to curing HIV infection aims at inducing viral expression using latency-reversing agents (LRAs) to allow the elimination of infected cells. Here, we tested a combination of two recently identified LRAs, the SMAC mimetic/IAP inhibitor AZD5582, which activates the noncanonical NF-κB pathway, and the antibody (Ab) MT807R1, which depletes CD8α^+^ cells, in SIV-infected rhesus macaques (RMs) on ART.

## INTRODUCTION

CD4^+^ T cells carrying integrated replication-competent human immunodeficiency virus (HIV) are recognized as the major barrier to the eradication of HIV in individuals on long-term antiretroviral therapy (ART) (1–3). Chief among the strategies being pursued toward eliminating persistent infection are approaches seeking to induce HIV expression within latently infected cells using latency-reversing agents (LRAs), followed by clearance of these cells directed by the host immune response or by immunotherapeutic interventions (4, 5). LRA candidates tested in clinical trials to date, such as protein kinase C modulators and histone deacetylase inhibitors, have been associated with limited efficacy (6–11). However, we recently identified a new class of LRA, targeting the noncanonical NF-κB (ncNF-κB) pathway, that is able to induce viral reactivation *in vivo*. Using a small molecule that induces the degradation of inhibitor of apoptosis protein (IAP; also known as the second mitochondrial activator of caspases [SMAC]), AZD5582, in rhesus macaques (RMs) infected with simian immunodeficiency virus (SIV) and in humanized mice infected with HIV, we demonstrated latency reversal on ART, as defined by increased viral RNA levels (12). Treatment with AZD5582 induced on-ART viremia in both animal models, SIV RNA expression in resting CD4^+^ T cells from lymphoid tissues, and HIV RNA expression from almost every tissue analyzed in humanized mice.

Several factors influence HIV latency establishment and maintenance, and recent reports suggest that CD8^+^ T cells may contribute to this process (13–15). Indeed, while HIV-specific cytotoxic T lymphocytes have been known for decades to modulate virus replication (16–21), more-recent studies suggest that CD8^+^ T cells may also suppress viral transcription during ART (13, 14, 22). In three independent *in vivo* studies, two performed on SIV-infected, ART-suppressed RMs and one on HIV-infected bone marrow-liver-thymus (BLT) humanized mice treated with ART, we demonstrated that experimental CD8α^+^ cell depletion was consistently followed by increases in plasma viral loads (13, 14). Phylogenetic analysis of the rebounding virus in these *in vivo* studies, as well as *ex vivo* work, suggests a key role for noncytolytic mechanisms silencing virus transcription, thus contributing to latency establishment and maintenance (14, 22). Furthermore, experimental CD8α^+^ cell depletion revealed the latency-reversing activity of the interleukin 15 (IL-15) superagonist N-803, which was not seen when N-803 was used alone, as shown by on-ART viremia and increased SIV RNA in tissues. This study suggested that CD8^+^ T cells might inhibit latency reversal during an HIV cure approach (14).

In the current study, we tested the hypothesis that CD8^+^ T cells contribute to latency maintenance by combining experimental CD8α^+^ cell depletion with AZD5582 treatment in SIV-infected, ART-suppressed RMs. This *in vivo* study included six SIV-infected RMs in which virus replication was effectively suppressed with a potent three-drug ART regimen for 84 to 85 weeks before additional interventions. We compared latency reversal induced by AZD5582 treatment alone (12), antibody (Ab)-induced depletion of CD8α^+^ cells alone (14), and a combination of both, and we used mathematical modeling to further interrogate the role of CD8^+^ T cells in viral latency during ART.

## RESULTS

### Experimental design.

**FIG 1:**
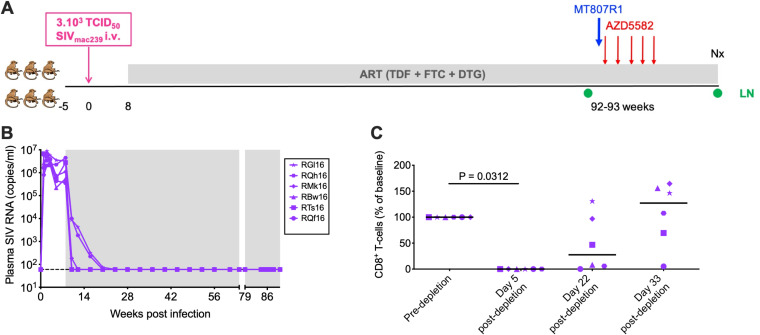
Experimental design. (A) Six RMs were infected i.v. with 3,000 TCID_50_ of SIV_mac239_. Starting at week 8 p.i., the RMs received ART daily. After 84 to 85 weeks of ART, all RMs received a single dose of the CD8α-depleting antibody MT807R1 at 50 mg/kg s.c., followed by five weekly doses of AZD5582 starting 1 day after MT807R1 administration. AZD5582 was infused i.v. at 0.1 mg/kg. All six RMs were sacrificed 10 to 17 days after the last dose of AZD5582. Lymph nodes (LN) were collected preintervention (28 days before MT807R1 administration) and postintervention, at necropsy (Nx) (days 42 to 58 after MT807R1 administration). (B) Plasma SIV RNA levels in the six RMs prior to CD8α^+^ cell depletion and treatment with AZD5582. Shading represents the period of ART administration. (C) The percentages of peripheral CD8β^+^ T cells relative to the predepletion baseline were calculated at days 5, 22, and 33 after MT807R1 administration.

We assessed *in vivo* the impact of Ab-mediated CD8α^+^ cell depletion on SIV latency reversal induced by the activation of the ncNF-κB pathway. Six male Indian RMs were infected intravenously (i.v.) with 3 × 10^3^ 50% tissue culture infective doses (TCID_50_) of SIV_mac239_. Starting at day 56 postinfection (p.i.), all six animals were initiated on triple ART consisting of two reverse transcriptase inhibitors (tenofovir [TDF] and emtricitabine [FTC]) and one integrase inhibitor (dolutegravir [DTG]). After 84 to 85 weeks on ART and sustained plasma viral load suppression to <60 SIV RNA copies/ml, the RMs received one dose of the CD8-depleting Ab MT807R1 (anti-CD8α) at 50 mg/kg of body weight subcutaneously (s.c.), followed by five weekly i.v. infusions of AZD5582 at 0.1 mg/kg (Fig. 1A and B). We selected a 5-dose design for AZD5582 administration to assess latency reversal during the period of maximal peripheral blood CD8α^+^ cell depletion based on our prior work (13, 14). As described previously (13, 14), using a single dose of MT807R1, we were able to deplete >99% of CD8^+^ T cells in peripheral blood by day 5 after administration of the depleting Ab. Partial and variable restoration of CD8^+^ T cells was observed in all treated RMs by day 33 after antibody administration (Fig. 1C). Three additional groups of animals served as controls: 4 RMs maintained on ART only (12), 9 RMs that received AZD5582 only (10 i.v. infusions at 0.1 mg/kg) (12), and 14 RMs that received CD8α^+^ cell depletion only (a single s.c. dose of MT807R1 at 50 mg/kg) (14). The details of the viral challenge (virus, dose, route), the ART regimen, the time of ART initiation postinfection, MT807R1 doses, and AZD5582 doses were identical for all the groups.

### AZD5582 treatment induces increases in on-ART viremia in 100% of CD8α-depleted, ART-suppressed RMs.

**FIG 2:**
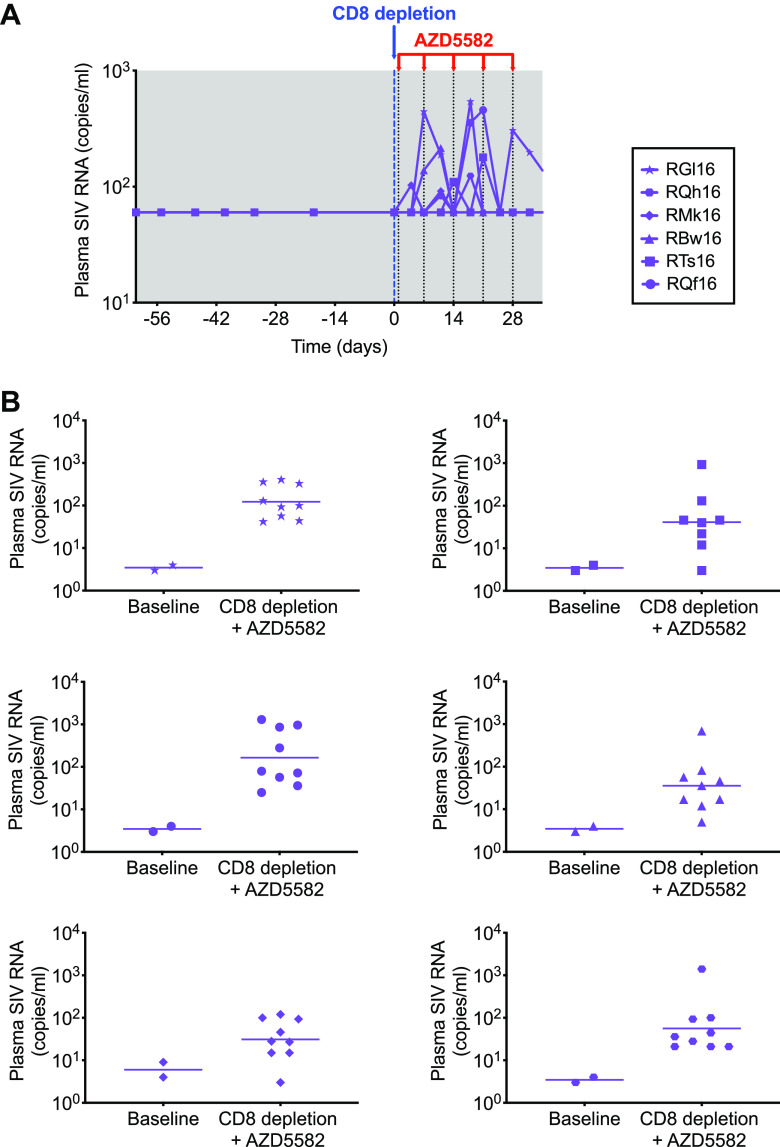
SIV reactivation with AZD5582 following CD8α^+^ cell depletion. (A) Plasma SIV RNA levels in SIV-infected, ART-suppressed RMs during AZD5582 treatment and CD8α cell depletion, determined by a standard viral load assay with a limit of detection of 60 copies/ml of plasma. Shading represents the period of ART administration. (B) Individual representations of plasma SIV RNA levels measured by an ultrasensitive plasma viral load assay (limit of detection, 3 copies/ml of plasma) before and during the combined CD8α depletion–AZD5582 treatment in SIV-infected, ART-suppressed RMs.

To assess latency reversal, we quantified plasma viral loads before and 4 days after each dose of AZD5582 using both a standard assay with a limit of detection of 60 copies/ml of plasma and an ultrasensitive assay with a limit of detection of 3 copies/ml of plasma. As shown in Fig. 2A, all six SIV-infected RMs showed increases in viremia to >60 copies/ml of plasma after combined CD8α^+^ cell depletion and AZD5582 treatment. This increased viremia was observed in one RM at the first time point assessed, 4 days after the first dose of AZD5582 (5 days after CD8α cell depletion). After the third dose of AZD5582, all six RMs had experienced at least one episode of viremia at >60 copies/ml. We next used an ultrasensitive assay to compare plasma viral loads at two baseline time points during ART within the 4 weeks preceding CD8α^+^ cell depletion and AZD5582 treatment (when plasma SIV RNA was <60 copies/ml in all RMs as determined by the standard viral load assay) and at eight to nine time points during CD8α^+^ cell depletion and AZD5582 treatment. Each RM showed multiple measurements above their individual baseline median during CD8α^+^ cell depletion and AZD5582 treatment (Fig. 2B).

### AZD5582-induced viral reactivation is enhanced by experimental CD8α^+^ cell depletion.

**FIG 3:**
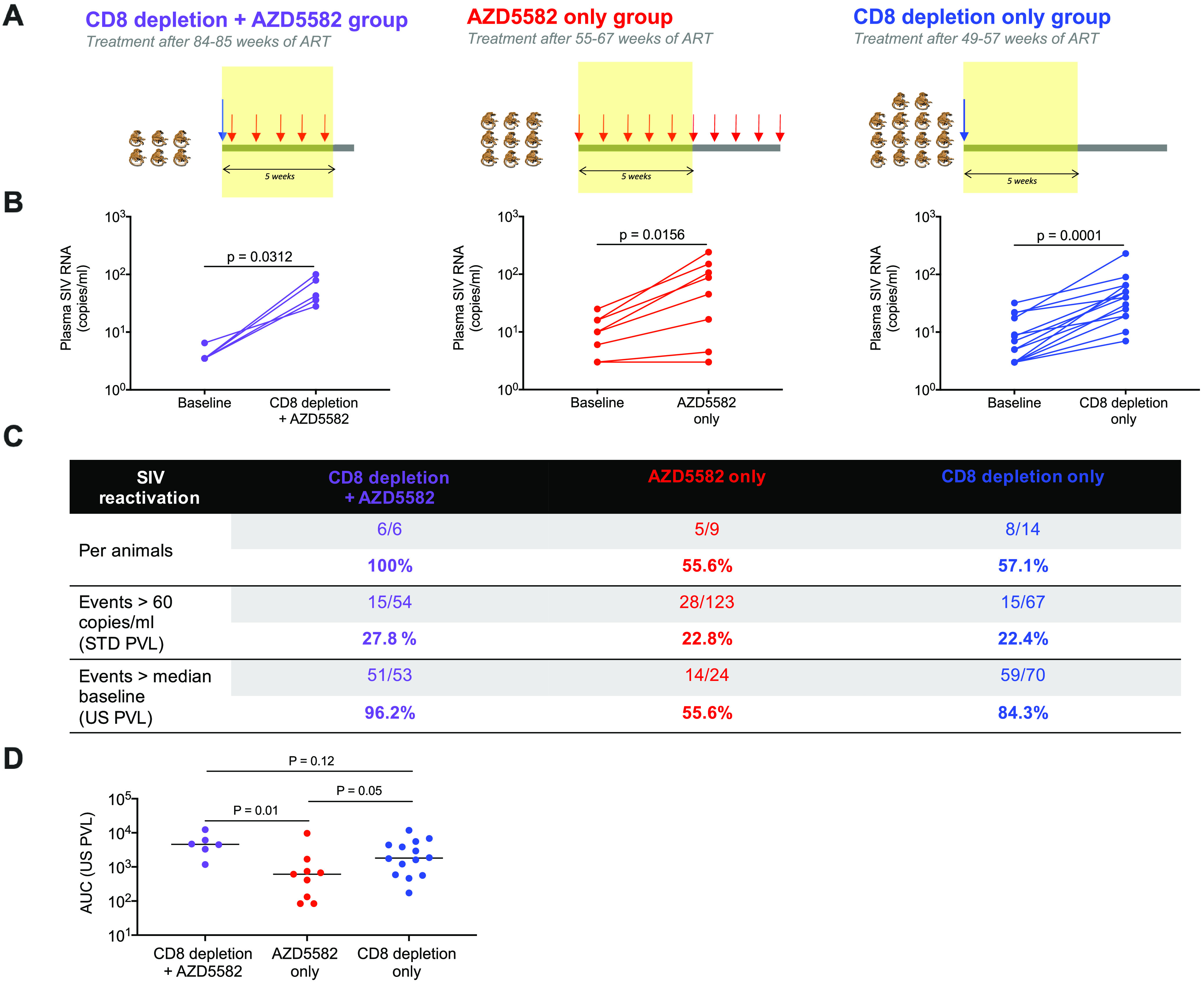
Characterization of on-ART viremia. (A) Intervention period studied for three groups of SIV-infected, ART-suppressed RMs: those receiving combined CD8α depletion and AZD5582, AZD5582 only, or CD8α depletion only. A group of 6 RMs treated with 5 weekly doses of AZD5582 after CD8α^+^ cell depletion was compared to a group of 9 RMs that received AZD5582 only, with comparisons made during the first 5 weekly doses of AZD5582 (of 10 total), and to a group of 14 RMs that received the CD8α^+^ cell-depleting antibody MT807R1 only, with comparisons made during an equivalent 5-week period following antibody administration. (B) Comparison of the medians of baseline plasma SIV RNA levels and levels during intervention, measured by an ultrasensitive assay, for SIV-infected, ART-suppressed RMs. “Baseline” represents the medians of plasma viral loads before intervention, and “CD8α depletion + AZD5582,” “AZD5582 only,” or “CD8α depletion only” represents the median plasma viral loads during 5 weeks following intervention. Statistical significance was determined with a Wilcoxon matched-pairs signed-rank test. (C) Comparisons of SIV reactivation during the intervention period, as defined by on-ART increased plasma viral loads, between CD8α depletion-plus-AZD5582, AZD5582-only, and CD8α depletion-only groups of SIV-infected, ART-suppressed RMs. STD, standard assay; PVL, plasma viral load; US, ultrasensitive assay. (D) Comparison of areas under the curve for US PVL data during the intervention period from CD8α depletion-plus-AZD5582, AZD5582-only, and CD8α depletion-only groups of SIV-infected, ART-suppressed RMs.

To further evaluate the role of CD8^+^ T cells in viral latency maintenance on ART, we compared treatment-associated increases in plasma SIV RNA levels in the RMs treated with the CD8-depleting antibody before AZD5582 treatment to those for a group of 9 animals that received AZD5582 only and a group of 14 animals that received MT807R1 only (Fig. 3A). The virus inoculum, infectious dose, infection route, time of ART initiation postinfection, and ART regimen were identical for the three groups of animals. The AZD5582 dose was also the same for all groups. Although the nine RMs that were treated with AZD5582 alone received 10 doses, for the comparisons included here we utilized only data obtained during the first 5 doses, to match the AZD5582 dosing received by the CD8α depletion-plus-AZD5582 group. It should also be noted that the RMs that received AZD5582 only or CD8α depletion only were on ART for 55 to 67 weeks or 47 to 59 weeks, respectively, at the time the first dose was administered, whereas the CD8α depletion-plus-AZD5582 group received ART for 84 to 85 weeks. While we observed increases in plasma viral loads to >60 copies/ml in 56% of the RMs treated with AZD5582 only and 57% of the RMs treated with MT807R1 only, 100% of the RMs from the CD8α depletion-plus-AZD5582 group experienced on-ART viremia of >60 copies/ml during the AZD5582 treatment period. We also utilized an ultrasensitive plasma viral load assay (limit of detection, 3 copies/ml) to study latency reversal in these three groups. The median levels of SIV RNA in plasma were significantly higher during the intervention period than at baseline for all groups (Fig. 3B) (*P*, 0.0312 for the CD8α depletion-plus-AZD5582 group, 0.0156 for the AZD5582-only group, and 0.0001 for the CD8α depletion-only group). We also found that 96% of the measurements were above baseline in the CD8α depletion-plus-AZD5582 group versus 56% in the AZD5582-only group and 84% in the CD8α depletion-only group (Fig. 3C), despite the longer period of ART suppression in the combination group. We also compared the areas under the curve (AUC) for the plasma viral load data from the ultrasensitive assay between the three groups of animals (Fig. 3D). The median AUC values were ranked as follows: for the CD8α depletion-plus-AZD5582 group, 4,586.75; for the CD8α depletion-only group, 1,806.75; and for the AZD5582-only group, 612.5. The area under the curve for the plasma viral load data from the ultrasensitive assay was significantly higher in the CD8α depletion-plus-AZD5582 group than in the AZD5582-only group of SIV-infected, ART-suppressed RMs (*P* = 0.01). Overall, these results indicate that the latency reversal activity of AZD5582 is enhanced by experimental CD8α^+^ cell depletion, a conclusion consistent with a role for CD8^+^ T cells in the maintenance of HIV/SIV latency on ART.

**FIG 4:**
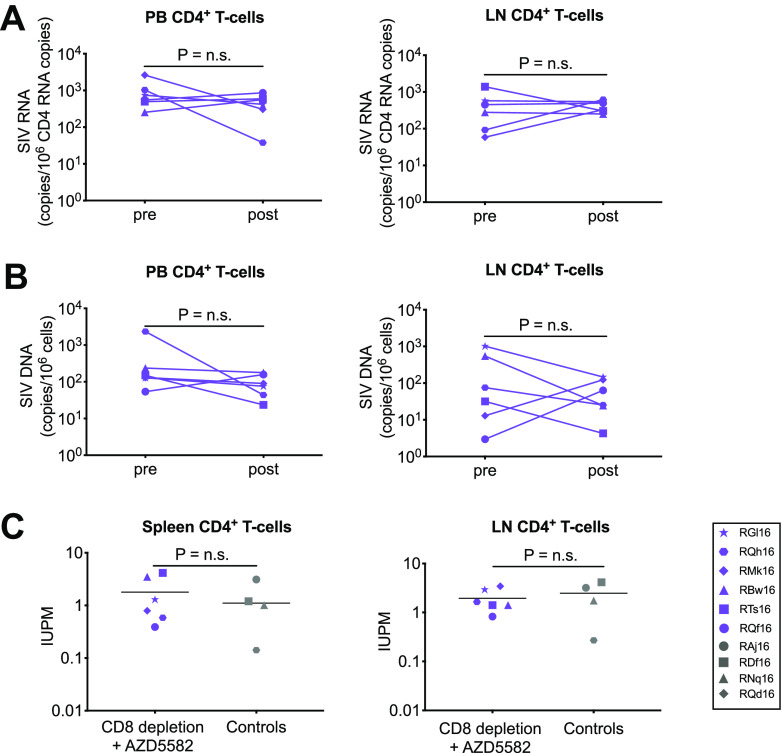
SIV reservoir in SIV-infected, ART-suppressed RMs. (A and B) Cell-associated SIV RNA (A) and DNA (B) levels in resting CD4^+^ T cells isolated from peripheral blood (PB) and lymph nodes (LN) of SIV-infected, ART-suppressed RMs before (pre) and after (post) CD8α^+^ cell depletion and 5 doses of AZD5582. Statistical significance was determined with a Wilcoxon matched-pairs signed-rank test. LN were collected preintervention (28 days before MT807R1 administration) and postintervention, at necropsy (days 42 to 58 after MT807R1 administration). PBMCs were collected preintervention (19 days before MT807R1 administration) and postintervention, at necropsy (days 42 to 58 after MT807R1 administration). n.s., not significant. (C) Comparison of replication-competent SIV levels in CD4^+^ T cells isolated from LN and spleens collected at necropsy between the combined CD8α depletion-and-AZD5582 group and control RMs receiving ART only. Quantitative viral outgrowth assays were performed for the CD8α depletion-plus-AZD5582 group after 84 to 87 weeks of ART followed by CD8α^+^ cell depletion and five doses of AZD5582, and for control RMs after 61 to 88 weeks of ART. IUPM, infectious units per million CD4^+^ T cells. Statistical significance was determined with a two-sided Mann-Whitney U test. Horizontal lines represent the medians.

Despite increased induction of on-ART viremia in the group receiving both CD8α depletion and AZD5582, no significant differences between the levels of total cell-associated RNA or DNA after AZD5582 treatment and baseline levels were observed in resting CD4^+^ T cells isolated from the peripheral blood or lymph nodes of CD8α-depleted RMs (Fig. 4A and B). We also estimated the levels of replication-competent virus by a quantitative viral outgrowth assay (QVOA) after CD8α^+^ cell depletion and AZD5582 treatment, and we compared these levels to those of controls that received a placebo after a similar time on ART (Fig. 4C). The levels of replication-competent SIV were similar in the two groups, suggesting that short-term treatment with AZD5582 did not significantly affect this measurement of persistent infection in CD8α-depleted RMs.

**FIG 5:**
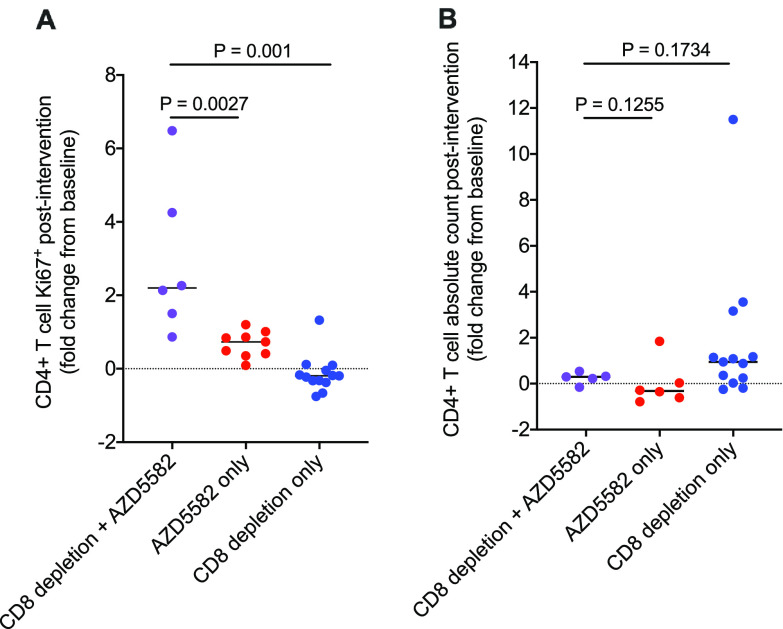
CD4^+^ T cell proliferation and absolute counts. Shown are levels of Ki67 expression in CD4^+^ T cells (A) and absolute counts of CD4^+^ T cells (B) in the peripheral blood of groups treated with CD8α depletion plus AZD5582, AZD5582 only, or CD8α depletion only. Data are expressed as the fold changes from baseline levels after intervention. Statistical significance was determined with a two-sided Mann-Whitney U test. Horizontal lines represent the medians.

Of note, we previously reported an increase in CD4^+^ T cell proliferation following AZD5582 treatment (in blood but not in lymph nodes, based on Ki67 expression) (12). This was also observed in the current study, with a higher fold change from the baseline in Ki67 expression in the CD8α depletion-plus-AZD5582 group than in the AZD5582-only and CD8α depletion-only groups (Fig. 5A). However, the CD4^+^ T cell counts after intervention were similar to the baseline counts in all groups, and the change in CD4^+^ T cell counts in the combined-treatment group was not significantly different from those in the other two groups (Fig. 5B). Further, we note that the level of CD4^+^ T cell-associated SIV DNA was stable following CD8α depletion and AZD5582 treatment, suggesting that this increase in Ki67 expression did not result in expansion of the pool of infected cells (Fig. 4).

### Mathematical modeling of AZD5582-induced viral reactivation supports the role of CD8^+^ T cells in viral latency maintenance on ART.

**FIG 6:**
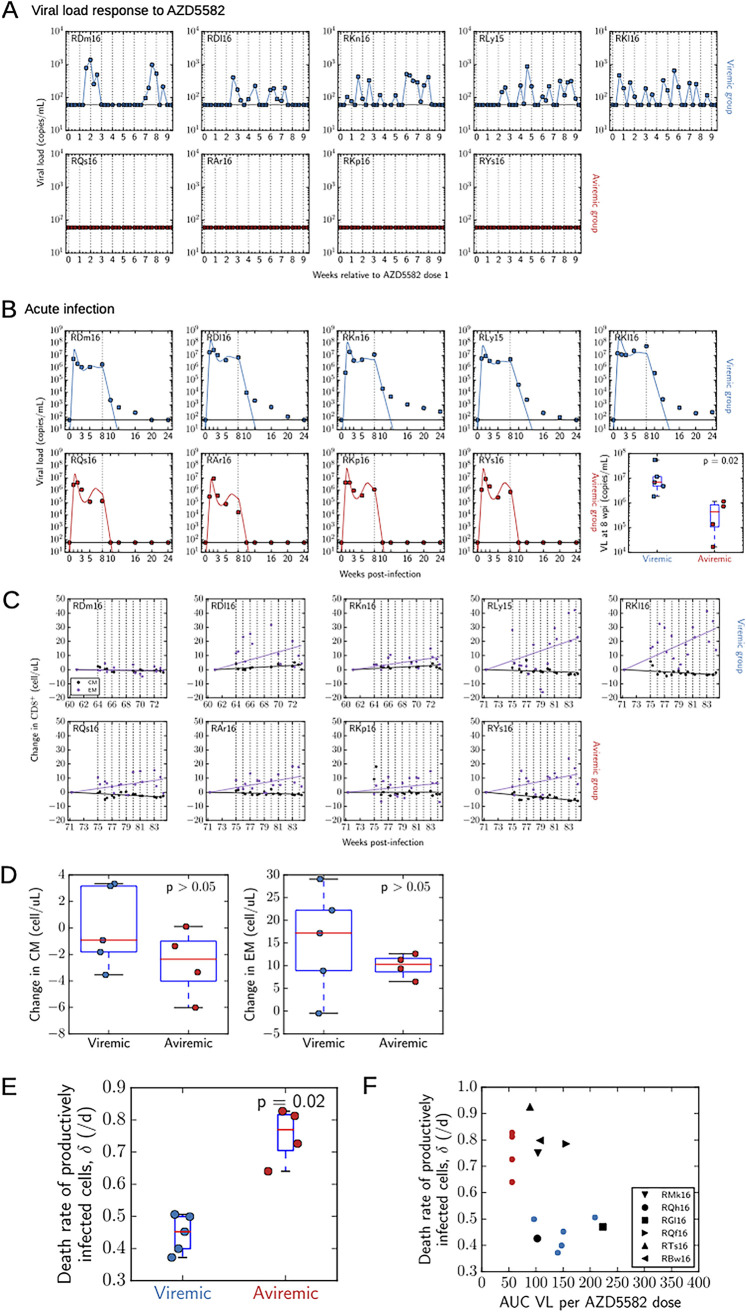
Mathematical modeling of acute SIV infection and response to AZD5582. (A and B) To build the model, plasma viral loads of >60 copies/ml (horizontal line) were assessed for the AZD5582-only group during AZD5582 treatment (all 10 doses) (vertical lines) (A) and during acute infection and the initiation of ART at 8 weeks postinfection (wpi) (vertical line) (B). RMs in the AZD5582-only group are segregated by their viral load responses to AZD5582 into viremic (blue) and aviremic (red) groups. Viral loads at the time of ART initiation (8 wpi) were compared between the animals in the viremic and aviremic groups, and statistical significance was determined with a two-sided Mann-Whitney U test. (C) Longitudinal central memory (CM) and effector memory (EM) CD8^+^ T cell counts during AZD5582 treatment (10 doses) (vertical lines) are shown for individual SIV-infected, ART-suppressed RMs in the AZD5582-only treatment group. (D) Changes in CM or EM CD8^+^ T cell counts from the baseline after AZD5582 treatment were compared between the viremic and aviremic groups. Statistical significance was determined with a two-sided Mann-Whitney U test. (E) Estimates of the death rate of productively infected cells were made via fits of a standard mathematical model of within-host HIV infection to the acute-infection viral load data up to 10 weeks postinfection (corresponding to the solid colored lines in panel B), and the viremic and aviremic groups were compared. Statistical significance was determined with a two-sided Mann-Whitney U test. (F) The estimate of the death rate of productively infected cells was associated with the viral load response to AZD5582, given as the area under the viral load curve (AUC VL) normalized to the number of AZD5582 doses. For the AZD5582-only treatment group, results for viremic RMs are shown as blue circles and results for aviremic RMs as red circles. The estimates for RMs in the combined CD8α depletion-plus-AZD5582 group are shown as black symbols, with two RMs aligning with the AZD5582-only viremic group and four RMs with the AZD5582-only aviremic group (although all six RMs in the combined CD8α depletion-plus-AZD5582 group demonstrated on-ART viremia during the intervention).

To further explore the role of CD8^+^ T cells in controlling viremia, we used mathematical modeling to explain viral load patterns observed in the nine RMs that received AZD5582 only (no CD8α^+^ cell depletion) and the six RMs that received both AZD5582 and the anti-CD8α Ab MT807R1. Among the nine RMs that received AZD5582 only, four did not show increases in plasma viral loads to >60 copies/ml (the aviremic group), while the remaining five RMs did (the viremic group). We found that these responses to AZD5582 (aviremic versus viremic) correlated with the viral load dynamics during acute infection. When RMs were grouped by their viral load responses to AZD5582 (Fig. 6A), differences in acute viremia and responses to ART were clear. The animals in the viremic group took longer to suppress viral loads once on ART, showing a slow biphasic decline in viral loads, than the RMs in the aviremic group, which rapidly suppressed plasma viral loads within the first 2 weeks on ART (Fig. 6B). We compared the plasma viral loads in these nine RMs during acute infection and found that the two groups achieved similar peak viral loads of 10^6^ to 10^7^ copies/ml (*P* > 0.05) but that the aviremic group had significantly lower viral loads at the time of ART initiation than the viremic group (means, 5 × 10^5^ versus 2 × 10^7^ copies/ml [*P* = 0.02]). This suggests that the RMs in the aviremic group were better able to control virus replication than the RMs in the viremic group and that this difference, already discernible during acute infection, persisted during ART. Assessment of the absolute counts of central memory (CM) and effector memory (EM) CD8^+^ T cells during AZD5582 treatment showed fluctuations in both the viremic and aviremic groups, with a slight trend in both groups for the EM population to expand (Fig. 6C). However, no statistically significant changes were observed between the baseline and the end of AZD5582 dosing when the viremic and aviremic groups were compared (Fig. 6D).

To characterize possible differences in the immune response between the animals in the viremic and aviremic groups, we used a variant of the standard mathematical model of within-host HIV infection (23) that included an eclipse phase and fitted the model to the acute-infection viral load values up to 10 weeks postinfection, from the time of SIV inoculation through the first 2 weeks of ART. Given the absence of significant changes in CD8^+^ T cell counts, we used a viral dynamic model that assumes that productively infected cells are killed at a constant rate (δ) per cell by a combination of natural death, viral cytopathic effects, and effector cell killing. We estimated the viral production rate, infection rate, and death rate of productively infected cells. Only the estimates of the death rate of productively infected cells differed significantly between the groups: the mean estimate for the aviremic group was almost two times higher than that for the viremic group (0.75/day versus 0.45/day [*P* = 0.02]) (Fig. 6E). In this model, the death rate of productively infected cells represents a combination of viral cytopathic effects, activation-induced cell death, natural death, and immune-mediated clearance.

When we associated the viral load response to AZD5582 (given by the area under the viral load curve) with the acute-infection dynamics (as summarized by the estimated death rate of productively infected cells), there was, again, a clear distinction between the aviremic and viremic groups (Fig. 6F, red and blue circles, respectively). A similar analysis of the acute-infection dynamics in the RMs in the CD8α depletion-plus-AZD5582 group was performed. The acute-infection viral load dynamics and responses to ART in four RMs in the CD8α depletion-plus-AZD5582 group (Fig. 6F, triangles) resembled those seen in the aviremic-group animals, while the dynamics in two RMs (Fig. 6F, black circle and square) resembled those seen in the viremic-group animals from the AZD5582-only study. Based on these acute-infection parameters, the four RMs (Fig. 6F, triangles) were thus predicted not to show increases in plasma viral loads of >60 copies/ml when given AZD5582. Yet during CD8α^+^ cell depletion and AZD5582 treatment, these four RMs did show on-ART viremia that was similar to that in the AZD5582-only viremic group. These results suggest that CD8α^+^ cell depletion influences the response to AZD5582, thus supporting a potential role for CD8^+^ T cells in maintaining viral latency during ART.

## DISCUSSION

Lifelong ART is associated with multiple issues, including stigma, side effects, and economic burden. Eradicating HIV from infected individuals thus remains a priority for HIV research, and as such, gaining a better understanding of the factors involved in HIV latency to develop new curative strategies is critical. Here, we tested *in vivo* a novel combination approach using the newly identified latency-reversing agent AZD5582 and experimental depletion of CD8α^+^ cells in SIV-infected, ART-suppressed RMs.

In agreement with prior findings, treatment with the anti-CD8α Ab MT807R1 depleted >99% of CD3^+^ CD8^+^ T cells in the peripheral blood of SIV-infected, ART-suppressed RMs. AZD5582 was then administered safely starting at day 1 post-CD8α depletion. This combined treatment was followed by sustained increases in plasma SIV RNA levels in all experimental animals. This result confirms the powerful virus reactivation induced by AZD5582 *in vivo* that was observed previously in SIV-infected, ART-suppressed RMs and in ART-suppressed BLT humanized mice (12).

Of note, viremia of >60 copies/ml was observed in 100% of the animals from the CD8α depletion-plus-AZD5582 group, as opposed to 56% in a group of 9 SIV-infected, ART-suppressed RMs treated with AZD5582 only and 57% in a group of 14 SIV-infected, ART-suppressed RMs treated with CD8α depletion only. While our groups of animals were matched in terms of viral challenge (virus, dose, route), the ART regimen, and the time of ART initiation postinfection, it should be noted that RMs from the CD8α depletion-plus-AZD5582 group were on ART up to 30 weeks longer than the RMs that received AZD5582 only. This additional time on ART potentially led to a qualitatively different reservoir, less prone to viral reactivation. As such, our comparison might underestimate the role of CD8^+^ T cells in suppressing the latency reversal activity of AZD5582. Despite this difference, increased levels of SIV RNA during AZD5582 treatment were more frequently observed in the CD8α depletion-plus-AZD5582 group, suggesting that CD8α^+^ cell depletion potentiates the ability of AZD5582 to induce on-ART viremia. Similarly, we have shown previously that administration of the IL-15 superagonist N-803 to both SIV-infected macaques and HIV-infected humanized mice induced a highly robust and persistent increased viremia only in the setting of CD8α^+^ cell depletion (14). We have thus now shown in two independent studies, the current study with AZD5582 and the N-803 study (14), an enhanced ability of two different LRA compounds to cause clinically relevant on-ART viremia in the absence of CD8^+^ cells. In line with previous studies showing that experimental CD8α^+^ cell depletion induced viremia during short-term and long-term ART, these results suggest a role for CD8^+^ T cells in maintaining HIV/SIV latency.

It is important to consider all potential effects of the depleting Ab used in this work, MT807R1. Since this Ab is directed at the CD8α chain, it also depletes CD3^−^ CD8α^+^ NK cells or T cell receptor (TCR) γδ^+^ T cells in addition to CD8α^+^ T cells. We have shown previously that following treatment with MT807R1, the depletion and repopulation of CD8^+^ T cells, but not CD3^−^ CD8α^+^ NK cells, is temporally associated with the control of viremia under ART (13). We also have evidence that treatment of ART-suppressed RMs with a CD8β Ab that targets CD8^+^ T cells but not NK or γδ^+^ T cells in combination with N-803 results in latency reversal (24). This particular Ab was not used in the present study due to its known inefficiency in depleting cells with a single dose, since our objective was to observe the impact of high-level CD8^+^ T cell depletion just prior to AZD5582 treatment. MT807R1 is also less effective at depleting CD8^+^ T cells in tissues than in blood; however, we note studies that have demonstrated that CD8^+^ T cells are functionally impaired following binding of the anti-CD8 Ab even if they are not physically depleted (25).

The precise mechanisms involved in CD8^+^ T cell control of HIV/SIV latency are not fully elucidated yet. Experimental depletion of CD8α^+^ cells has been associated with moderate CD4^+^ T cell homeostatic proliferation (13, 26, 27), raising the possibility of an indirect effect of the depletion mediated by increased availability of certain cytokines, such as IL-15, for CD4^+^ T cells. This indirect effect would then involve increased infected CD4^+^ T cell proliferation and activation rather than a direct antiviral effect of CD8^+^ T cells. While we observed an increase in CD4^+^ T cell proliferation following CD8α^+^ cell depletion and AZD5582 treatment, this did not translate into an increase in the CD4^+^ T cell count. Instead, several lines of evidence suggest a direct effect of CD8^+^ T cell depletion on latency reversal. First, we showed previously in SIV-infected, ART-treated RMs that the strong homeostatic proliferation that follows Ab-mediated CD4^+^ T cell depletion did not induce on-ART viremia (28). Second, treatment of SIV-infected, ART-suppressed RMs with the IL-15 superagonist (N-803) that causes CD4^+^ T cell activation and proliferation did not result in latency reversal (14). Third, the effects of N-803 on gene expression and the CD4 proliferative response were similar in the presence and absence of CD8^+^ T cells, yet virus reactivation occurred only when CD8^+^ T cells were depleted (14). Finally, viral production induced by IL-15 or N-803 from latently infected CD4^+^ T cells is inhibited by the addition of CD8^+^ T cells *in vitro* (14).

In the current study, mathematical modeling of viral reactivation induced by AZD5582 in RMs that received or did not receive the CD8α-depleting Ab suggests that the extent of viral reactivation observed during AZD5582 treatment is influenced not only by the ability of the animals to control viremia during acute infection but also by the CD8α-depleting Ab treatment. The mechanisms by which CD8^+^ T cells might control HIV/SIV latency are under investigation in our laboratories. We recently demonstrated that CD8^+^ T cells effectively suppress HIV transcription *ex vivo*, using CD4/CD8 coculture assays. Specifically, we found that non-HIV-specific, TCR-activated CD8^+^ T cells potently suppress HIV transcription via a noncytolytic, non-major histocompatibility complex (MHC)-restricted immunoregulatory mechanism that reduces CD4^+^ T cell activation and promotes the survival of latently infected CD4^+^ T cells (22). Further investigations of this mechanism could inform the development of new therapeutic strategies aimed at curing HIV.

This study advances our understanding of HIV/SIV latency during ART by highlighting the potential of CD8^+^ T cells to inhibit latency reversal. While intense research focuses on developing “shock and kill” strategies, it is critical to appreciate all the factors involved in latency maintenance. Modulation of the pathways that CD8^+^ T cells may use to promote latency could be key in achieving a cure for HIV infection.

## MATERIALS AND METHODS

### Ethics statement.

All animals were housed at the Yerkes National Primate Research Center (Atlanta, GA) and treated in accordance with Emory University and Yerkes National Primate Research Center Institutional Animal Care and Use Committee (IACUC) regulations (PROTO201800308). All animal accesses were performed under anesthesia, and the animals were monitored for clinical well-being and for potential signs of pain and distress, which were alleviated with analgesics as needed. The animals were euthanized at the end of the study using methods consistent with recommendations of the American Veterinary Medical Association (AVMA) Panel on euthanasia and per the recommendations of the IACUC. Specifically, the animals were anesthetized with ketamine before i.v. administration of 100 mg/kg of pentobarbital.

### SIV infection.

Six male Indian rhesus macaques, with the exclusion of MamuB*08^+^ and MamuB*17^+^ animals, were included in this study and infected intravenously with 3 × 10^3^ TCID_50_ (50% tissue culture infectious doses) of SIV_mac239_ (*nef* open). SIV_mac239_ was titrated *in vitro* for viral infectivity by standard endpoint titration on CEMx174 cells. The TCID_50_ was calculated using a previously published method (29).

### Treatment.

All six rhesus macaques were treated with a potent three-drug ART regimen initiated 56 days after infection that consisted of two reverse transcriptase inhibitors, tenofovir disoproxil fumarate (5.1 mg/ml) and emtricitabine (40 mg/ml), plus the integrase inhibitor dolutegravir (2.5 mg/ml). ART was administered once daily at 1 ml/kg of body weight via the subcutaneous route. All six rhesus macaques were treated with MTR807R1 and AZD5582. One dose of MT807R1 was administered subcutaneously at 50 mg/kg. AZD5582 was infused weekly intravenously at 0.1 mg/kg starting a day following MTR807R1 treatment. RMs received five doses of AZD5582 and were euthanized 14 to 17 days after the last dose.

### Sample collection and processing.

EDTA-anticoagulated blood samples were collected regularly and were used for a complete blood count, routine chemical analysis, and immunostaining, with plasma separated by centrifugation within 1 h of phlebotomy. Peripheral blood mononuclear cells (PBMCs) were prepared by density gradient centrifugation. Lymph nodes were collected before (28 days before MT807R1 administration) and after (days 42 to 58 after MT807R1 administration) AZD5582 treatment. Spleens were collected postmortem. After two washes in RPMI medium and the removal of connective and fat tissues, tissues were ground using a 70-μm cell strainer. The cell suspensions obtained were washed and either immediately used for immunostaining or cryopreserved at −80°C until use.

### Historical animal groups.

Data from three additional groups of SIV-infected, ART-suppressed RMs were used for comparative analyses, including 14 RMs treated with MT807R1 only (14), 9 RMs treated with AZD5582 only (12), and 4 RMs on ART only (12). Viral challenge (virus, dose, route), the ART regimen and the time of ART initiation postinfection, MT807R1 doses, and AZD5582 doses were identical for all groups.

### Immunophenotyping by flow cytometry.

Multicolor flow cytometric analysis was performed on whole blood using predetermined optimal concentrations of the following fluorescently conjugated monoclonal antibodies: CD3-allophycocyanin (APC)-Cy7 (clone SP34–2), Ki-67-AF700 (clone B56), CCR7-fluorescein isothiocyanate (FITC) (clone A20), CD45RA-phycoerythrin (PE)-Cy7 (clone 5H9), and CD62L-PE (clone SK11) from BD Biosciences; CD8-Brilliant Violet 711 (BV711) (clone RPA-T8), CD4-BV650 (clone OKT4), and CD95-BV605 (clone DX2) from BioLegend; and CD28-PE-Cy5.5 (clone CD28.2) from Beckman Coulter. Flow cytometric acquisition and analysis of samples were performed on at least 100,000 events on an LSR II flow cytometer driven by the FACSDiva software package (BD Biosciences). Analyses of the acquired data were performed using FlowJo software (version 10.0.4; Tree Star).

### Total and resting CD4^+^ T cell enrichment.

Total CD4^+^ T cells were enriched using magnetic beads and column purification (Miltenyi Biotec) from the peripheral blood, lymph node, and spleen cells. Enriched CD4^+^ T cells were then stained with previously determined volumes of the following fluorescently conjugated antibodies: CD3-AF700 (clone SP34-2), CD8-APC-Cy7 (clone SK1), CD69-PE-CF594 (clone FN50), HLA-DR peridinin chlorophyll protein (PerCP)-Cy5.5 (clone G46-6) (all from BD Biosciences), and CD4-BV650 (clone OKT4) and CD25-PE-Cy7 (clone BC96) (both from BioLegend). Resting CD4^+^ T cells were defined as CD3^+^ CD4^+^ CD8^−^ CD69^−^ CD25^−^ HLA-DR^−^. Sorting was performed on a FACSAria LSR II system (BD Biosciences) equipped with FACSDiva software.

### Plasma RNA and cell-associated DNA and RNA viral quantification.

Plasma SIV_mac239_ loads were quantified regularly throughout the study and twice per week during the AZD5582 treatment period in the Translational Virology Core Laboratory of the Emory Center for AIDS Research using a standard quantitative PCR (qPCR) assay (limit of detection, 60 copies per ml of plasma) as described previously (30). Ultrasensitive quantification of plasma SIV_mac239_ loads (limit of detection, 3 copies per ml of plasma) was performed for two time points before AZD5582 treatment and eight to nine time points during AZD5582 treatment as described previously (31). Cell-associated SIV RNA and DNA were measured simultaneously in resting CD4^+^ T cells isolated from peripheral blood, spleen, bone marrow, and lymph nodes, lysed in buffer RLT Plus (Qiagen), and stored at −80°C. Nucleic acids were extracted using the AllPrep DNA/RNA minikit (Qiagen) according to the manufacturer’s recommendations with an on-column DNase digestion step. Cell-associated SIV_mac239_ *gag* DNA was quantified by qPCR using a 5′ nuclease (TaqMan) assay with SIV *gag* primers, and results were normalized to the RM albumin gene, as described previously (12). For the quantification of cell-associated RNA, RNA was reverse transcribed using the High-Capacity cDNA Reverse Transcription kit (Thermo Scientific) and random hexamers. SIV *gag* and the RM CD4 gene were quantified by qPCR of the resultant cDNA using TaqMan Universal Master Mix II (Thermo Scientific). The CD4 primer and probe sequences were as follows: Rh-CD4-F, 5′-ACATCGTGGTGCTAGCTTTCCAGA-3′; Rh-CD4-R, 5′-AAGTGTAAAGGCGAGTGGGAAGGA-3′; Rh-CD4 probe, 5′-AGGCCTCCAGCACAGTCTATAAGAAAGAGG-3′. The means from two replicate wells were used in all analyses.

### SIV quantitative viral outgrowth assay.

Replication-competent SIV reservoirs were measured by the Viral Reservoir Core Laboratory of the Emory Center for AIDS Research. Latently infected cells were quantified using a limiting dilution culture assay in which CD4^+^ T cells enriched from lymph node or spleen cells collected at necropsy by using magnetic beads and column purification (Miltenyi Biotec) were cocultured with CEMx174 cells in 5-fold serial dilutions ranging from 5 × 10^6^ cells per well to 4 × 10^5^ cells per well. The cells were cultured in RPMI medium containing 10% fetal bovine serum (FBS) and 100 U/ml IL-2 (Sigma). The ratio of target cells added was 4:1 for the two highest dilutions. A constant number of 1 × 10^6^ CEMx174 cells was added to all other wells. The cultures were split every 7 days, and fresh medium was added. After 21 days, the growth of virus was detected by reverse transcription-quantitative PCR (qRT–PCR). SIV RNA was isolated from 400 μl of the culture supernatant using the Zymo viral RNA isolation kit (Zymo Research). DNase treatment was performed using an RQ1 RNase-free DNase kit (Promega). A one-step qRT–PCR targeting SIV *gag* was performed using an Applied Biosystems 7500 real-time PCR system (Applied Biosystems) and the TaqMan Fast Virus 1-Step Master Mix (Thermo Scientific) for qRT–PCR with the following primers and probe: SIVgagFwd, 5′-GCAGAGGAGGAAATTACCCAGTAC-3′; SIVgagRev, 5′-CAATTTTACCCAGGCATTTAATGTT-3′; SIVgag probe, 5′–6-carboxyfluorescein (FAM)–TGTCCACCTGCCATTAAGCCCGA–Iowa black fluorescent quencher (IBFQ)–3′. The frequencies of infected cells were determined by the maximum-likelihood method (32) and were expressed as infectious units per million CD4^+^ T cells.

### Statistical analysis.

Statistical analyses were performed using GraphPad Prism software (version 8). A *P* value of 0.05 or less was considered statistically significant. The area under the curve with respect to ground was calculated as described previously using a method based on the trapezoidal rule (33).

### Mathematical modeling of acute SIV infection.

To analyze the plasma viral load dynamics during acute SIV infection, we used a standard mathematical model of within-host HIV/SIV infection that is described by the following set of ordinary differential equations:

$$\frac{\mathrm{dT}}{\mathrm{dt}}=\text{λ}-\mathrm{dT}-\hat{\beta }\mathrm{TV}$$

$$\frac{\mathrm{dE}}{\mathrm{dt}}=\hat{\beta }\mathrm{TV}-\mathrm{kE}$$

$$\frac{\mathrm{dI}}{\mathrm{dt}}=\mathrm{kE}-\mathrm{\delta I}$$

$$\frac{\mathrm{dV}}{\mathrm{dt}}=\mathrm{pI}-\mathrm{cV}$$

In this model, target cells (*T*) are supplied at rate λ and die at per capita rate *d*. Target cells can be infected by virus (*V*) with an effective infection rate equal to the infection rate constant ($\hat{\beta }=\text{β}$) and enter an eclipse phase, where they remain infected but are not yet producing virus for a mean duration of 1/*k*. Eclipse-phase cells (*E*) then become productively infected cells (*I*), which produce virus at rate *p* per cell for a mean duration of 1/δ before they die, are transcriptionally silenced, or are killed by a cell-mediated immune response. Virus is cleared at per capita rate *c*. To simulate the effect of ART, we assume that ART blocks viral infection with efficacy ε, where ε ranges between 0 (0% effective) and 1 (100% effective). Under ART, the effective infection rate is modified to $\hat{\beta }=\left(1-\text{ε}\right)\text{β}$.

We fitted the model to plasma viral load data from the acute infections of the nine RMs in the AZD5582-only group and the six RMs in the CD8α depletion-plus-AZD5582 group. We used data from the start of infection up to 10 weeks postinfection in order to capture the viral load dynamics through the first phase of viral load decay on ART (Fig. 6B, solid lines). The goodness of fit was given by the sum of squared residuals between the log_10_ of the viral load data and the model prediction. If the model-predicted viral load at a given time point was below the limit of detection (60 copies/ml), the residual was considered zero and the goodness of fit was not affected.

We fixed a number of parameters based on prior literature (Table 1) and estimated the remaining four parameters: the viral production rate (*p*), the basic reproductive number (*R*_0_), the death rate of productively infected cells (δ), and the initial virus concentration (*V*_0_). In order to directly estimate *R*_0_, the infection rate in the model was replaced by $\text{β}=\frac{R_{0}c\delta d}{p\lambda }$. Parameters were estimated using a differential evolution algorithm in Python (scipy.optimize.differential_evolution), and figures were created with phymcmc.plot (https://github.com/cbeauc/phymcmc).

**TABLE 1:** Fixed parameters in the acute-infection mathematical model

Parameter (abbreviation) (unit of measurement) (reference)	Value
Initial target cell conc (*T*_0_) (cells/ml)	10^6^
Target cell death rate (*d*) (/day) (34)	0.01
Target cell supply rate (λ = *dT*_0_) (cells/ml/day) (35)	10^4^
Eclipse-phase transition rate (*k*) (/day) (23)	1.1
Rate of loss of virus (*c*) (/day) (36)	23
Efficacy of ART (ε)	0.99
